# Influence of Grape Berry Maturity on Juice and Base Wine Composition and Foaming Properties of Sparkling Wines from the Champagne Region

**DOI:** 10.3390/molecules23061372

**Published:** 2018-06-06

**Authors:** Pin-He Liu, Céline Vrigneau, Thomas Salmon, Duc An Hoang, Jean-Claude Boulet, Sandrine Jégou, Richard Marchal

**Affiliations:** 1 Laboratory of Oenology, Université de Reims Champagne-Ardenne, Moulin de la Housse, BP 1039, 51687 Reims CEDEX 2, France; pinhe.liu@gmail.com (P.-H.L.); thomas.salmon@univ-reims.fr (T.S.); hoangducan@qnu.edu.vn (D.A.H.); sandrine.jegou@univ-reims.fr (S.J.); 2 Institut Œnologique de Champagne, 9 Rue du Commerce, 51350 Cormontreuil, France; cvrigneau@ioc.eu.com; 3 Institut National de la Recherche Agronomique, Unité Mixte de Recherches No. 1083, Sciences pour l’Œnologie, 2 Place Pierre Viala, 34060 Montpellier, France; jean-claude.boulet@inra.fr

**Keywords:** grape berry maturity, protein, foam, grape juice, base wine, sparkling wine, Pearson’s correlation

## Abstract

In sparkling wine cool-climate regions like Champagne, it is sometimes necessary to pick the healthy grape clusters that have a relatively low maturity level to avoid the deleterious effects of *Botrytis cinerea*. In such conditions, we know that classical oenological parameters (sugars, pH, total acidity) may change but there is little information concerning the impact of grape berry maturity on wine proteins and foaming properties. Therefore, healthy grapes (Chardonnay and Pinot meunier) in 2015 and 2016 were picked at different maturity levels within the range of common industrial maturity for potential alcohol content 8–11% v/v in the Champagne region. Base wine protein content and foamability, and oenological parameters in grape juice and their corresponding base wines, were investigated. The results showed that base wine protein contents (analyzed by the Bradford method and by electrophoresis) and foamability were higher when the grapes were riper. The Pearson’s correlation test found significant positive correlations (*r =* 0.890–0.997, *p <* 0.05) between Chardonnay grape berry maturity degree (MD) and base wine foamability in both vintages. Strong correlations between MD and most of the oenological parameters in grape juice and base wine were also found for the two cultivars. Under the premise of guaranteed grape health, delaying harvest date is an oenological decision capable of improving base wine protein content and foamability.

## 1. Introduction

Grape berry maturity is critical to control the quality of grape juice and wine and to determine harvest time. In the Champagne region, the potential alcohol content (PAC) of grape berries generally ranges from a minimum of 8% v/v (allowed in exceptional years after agreement between grape berry producers/cooperatives and the Champagne houses), to 11% v/v (value corresponding more to the personal decision of the winemaker). In fact, a juice sugar content of 135 g/L (or a PAC of 8% v/v) is not considered to have sufficient sensorial maturity [1]. However, it is accepted by the Champagne wine industry, because this low maturity could be compensated by blending with more mature wines from the same or previous vintages stored in stainless steel tanks for between one to four years [2]. Grape maturity varies with growing season variability from one year to the next. According to the Comité Interprofessionnel du Vin de Champagne (C.I.V.C.), the average PAC of ‘*Cuvée*’ grape juice was 9.4% v/v in both vintages 2007 and 2011, while 10.6% v/v in vintage 2012 and 10.5% v/v in 2015 [1]. Key differences in harvest dates have been observed for many years in the Champagne region. For instance, the beginnings of harvest (*les dates d’ouverture de vendanges*) in 2011, 2013 and 2015 were 23 August, 30 September and 8 September, respectively. Thus, a 38 days’ deviation from the start of harvest time has been recorded between the year 2011 and 2013. Furthermore, a sudden temperature drop accompanied by rainfall in Champagne can easily result in fungal infections. Owing to the heterogeneity of grape berries, *Botrytis cinerea* can more easily infect those riper grapes, due to their softer and/or more elastic skin and weaker defenses [3,4]. Under these circumstances, Champagne grape-growers have to advance the harvest dates to prevent *Botrytis cinerea* invading grapes, and to ensure the quality of grapes. Harvest dates in Champagne tend to be more dependent on the scale of fungal infections rather than the maturity degree of grape berries, which is more essential in warm areas.

Foam is an important organoleptic characteristic of sparkling wines, so research on Champagne and sparkling wines has predominantly focused on surface-active compounds and their relationships to foaming properties over the past few decades. Most of these studies indicate a positive correlation between protein content and foamability in grape juices [5] and wines [6,7,8]. Indeed, the content of soluble proteins in Gewürztraminer and Riesling grape juice and wine is positively correlated with the advance of grape berry maturity status [9], whilst the number of Muscadine wine proteins decreased from 1269 (green hard berries) to 1077 (ripe berries) [10]. However, there is little information concerning the impact of grape berry maturity on Champagne wine protein composition and content. Additionally, there is almost no literature regarding the possible relationship between grape berry maturity and the foaming properties of wine, a characteristic of prime importance for winemakers. Only one recent study [11] performed on Cava base and sparkling wines has discussed these correlations for four varieties of grapes (three Spanish varieties: Macabeo, Xarel.lo, Parellada and Chardonnay) which were harvested at two different maturity levels. Results showed that the Cava base and sparkling wines from the second harvest had higher protein concentrations but lower foamability.

In this study, three maturity levels of visually disease-free Chardonnay (CH) and Pinot meunier (PM) grapes and two maturity levels of disease-free Chardonnay grapes were harvested respectively in 2015 and 2016 in Champagne. The aim of this work was to study the relationships between grape maturity parameters (pH, sugars, total acidity, malic acid and grape berry MD) and Champagne base wine protein contents, foaming properties and oenological parameters.

## 2. Results and Discussion

The oenological parameters determined for PM and CH grape juices at different grape berry maturity levels and the corresponding base wines are shown in Table 1 and Table 2, respectively. Correlations between all these data were calculated using the Pearson test to find out if parameters were statistically correlated. Out of 1155 Pearson’s correlation coefficients (Table 3 and Table 4), 652 (60%) indicated significant or high correlations (*p* < 0.05) between two grape juice/base wine parameters under specific condition, such as grape variety (PM or CH), juice pressing cycle (squeeze 1 or 2) and year (2015 or 2016). These correlations can be classified as follows: 207 with *r* > 0.95, 178 with 0.95 > *r* > 0.8, 134 with *r* < −0.95 and 133 with −0.95 < *r* < −0.8. These results will be discussed in the following sections. We pay particular attention to the correlations that explain the impact of grape maturity on the wine protein contents and foamability.

### 2.1. Sugars and Acidity

Sugar content is a critical traditional indicator of grape berry maturity stage; it estimates PAC and is used (sometimes without other analytical measure) to decide the harvest date in winemaking industry. Between grape maturity level I and III, the sugar content of grape juices increased by 36% (PM 2015), 25% (CH 2015) and 21% (CH 2016) (Table 1 and Table 2). In 2015, an efficient sugar accumulation between maturity level I and II followed by a slow rise at level II–III was observed for both PM and CH grapes, which is in accord with the typical grape berry sugar evolution as previously reported [12,13]. From grape maturity level I to III, an increase and a decrease trend were respectively found in the pH and total acidity (TA) of grape juices and their corresponding base wines (Table 1 and Table 2). Grape juice TA declined with grape ripening mostly due to the catabolism of malic acid (MA) [14]. A greater loss of MA content (CH: −58%) was noted in 2016 when compared to the vintage 2015 (CH S1: −37%, CH S2: −35% and PM: −30%) from maturity level I to III (Table 1 and Table 2). It may be due to successive sunny days and no rain before the last sampling date (III) in 2016, thus accelerating grape maturation and the respiratory rate of berries [15,16].

Results also showed that grape juice tartaric acid (TartA) contents surprisingly increased for both cultivars in 2015 between the maturity level II and III. For the PM juice in 2015, for example, a decrease of the TartA content between the maturity level I (8.2 g/L) and II (7.0 g/L) was observed as expected. Nevertheless, a *Botrytis* contamination clearly appeared on a part of the bunches between the second (II) and the third (III) sampling date because of continuous rainy/humid days and relative low temperature allowing the fungus development. The best bunches, considered visually as not infected by *Botrytis*, were harvested for the last maturity point. In the 2015 PM grape juices, an increase of sugar content between the maturity level II (167.1 g/L) and III (182.9 g/L) was observed. This corresponded to a higher maturity for the winemaker (higher PAC % v/v). However, juice III was more acidic (higher TA) and richer in TartA content (9.4 g/L) than the juice II (8.2 g/L), whereas a decrease was expected. This is due to the selection of healthy bunches being less ripe. This problem was not observed for the CH 2016, for which the TartA content decreased by 1.5 g/L between the maturity levels I/II and III.

We have of course noted the presence of gluconic acid at very low concentrations in all 2015 wines, as well as in the 2016 wines (all of them produced from bunches visually sound and carefully selected). Nevertheless, these gluconic acid contents can be considered as very low when compared with a botrytized Chardonnay wine produced with bunches whose contamination level was visually estimated around 20% and containing 502 mg/L [17]. So, a *Botrytis* contamination could very slightly explain the increase of TartA contents and changes noted in the present study.

### 2.2. Maturity Degree

The grape berry MD is defined as the ratio sugars/TA. The highest values were observed for the third (last) sampling in both cultivars and vintages (Table 1 and Table 2). Table 2 also indicated distinct CH grape berry MD varying from 14 (I), 17 (II) to 23 (III) in 2015 (S1) and from 16 (I/II) to 28 (III) in 2016 (S1), respectively. This confirms that CH grapes were indeed harvested at different maturity levels. Nevertheless, PM grape berry MD remained unchanged between maturity level II and III in 2015, both reaching 21 for S1 and 23 for S2, respectively (Table 1). The explanation of this stability is the same as for the TartA content changes (selection of healthy bunches).

It is important to note that large differences are observed in the current literature for grape berry MD at harvest between regions with different/contrasting climates. For instance, 21 (PM) and 28 (CH) in Champagne region (cool climate), 29.5 for a Chardonnay and 41 for a Macabeo in Spain [11], 35 for a Chardonnay, 36.5 for a Merlot and 44 for a Syrah in South Australia (warm climate) [18]. These comparisons prove that the Champagne region juices have characteristics (sugars, pH, TA, TartA, MD) quite different from those generally observed in other regions producing sparkling wines. This will carefully be considered in the following sections dedicated to the study of proteins and foamability.

Strong positive correlations were determined between grape berry MD and pH value (*r =* 0.882–0.999), sugar content (*r =* 0.914–0.994) and PAC (*r =* 0.913–0.995) in grape juices and base wines (Table 3 and Table 4), whereas significant negative correlations were revealed between MD and TA (*r =* −0.979–−1.000) and MA (*r =* −0.973–−1.000), whatever the squeeze (S1 and S2), grape variety (CH and PM) or year (2015 and 2016). Concerning TartA of grape juices and base wines, no consistent correlation was found with grape berry MD during the final stage of ripening, which is consistent with previous studies [19,20] and the explanation given above.

### 2.3. Potassium and Calcium

Potassium (K^+^) is the most plentiful cation in grape berries and plays a crucial role in enzyme activation, osmotic potential regulation, and cation, anion and sugar uptake [21]. From maturity level I to III, the concentration of K^+^ (mg/L) in PM and CH grape juices increased by 3–9% in 2015 (Table 1 and Table 2). Table 3 and Table 4 show a positive correlation between grape juice K^+^ level and grape berry MD (*r =* 0.595–0.996) in 2015. With the increase of grape berry MD, K^+^ level strongly positively related to sugar content in PM S1 (*r =* 0.841), PM S2 (*r =* 0.846) and CH S2 (*r =* 0.940) grape juices in 2015. We cannot report the variation of K^+^ content in CH grape juices in 2016 due to the lack of data. However, a decrease of K^+^ level in CH base wines has been noted over the two grape maturity levels in 2016, which is contrary to the results in 2015. Comparing two varieties in 2015, PM grape juices had a higher K^+^ level (average 1328 mg/L) than CH (average 877 mg/L). These differences could be due to factors that influenced the uptake of K^+^ in grape berries, including soil, variety, vine microclimate and vineyard management [22].

The calcium (Ca^++^) concentration of CH and PM grape juices respectively decreased to approximately 30% and 20% from grape maturity level I to III in 2015 (Table 1 and Table 2). This decrease is mainly because of the large drop in xylem hydraulic conductance into the berry at veraison, and then due to dilution caused by berry enlargement during grape maturation [23]. It could be reinforced by significant negative correlations exposed between grape juice Ca^++^ level and MD (CH S1: *r =* −0.985, CH S2: *r =* −0.906, PM S1: *r =* −0.822 and PM S2: *r =* −0.890; Table 3 and Table 4).

### 2.4. Assimilable Nitrogen

Nitrogen is decisive in balance between vegetative and reproductive growth and predominantly stored in grape berries [24]. Results in Table 1 showed that the ammonium (NH_4_^+^), *α*-amino (*α*-NH_2_) and assimilable nitrogen (YAN) concentrations of PM grape juices in 2015 gradually increased from grape maturity level I to III. Nitrogen contents of CH grape juices in 2015 decreased from I to II and then strongly rose from II to III (Table 2), whereas their concentrations in CH grape juices in 2016 sharply decreased from maturity level I/II to III. In agreement with these results, paradoxical patterns in these nitrogen parameters during grape ripening were reported in previous studies [12,25,26]. In this study, high levels of nitrogen (CH/PM III 2015) tended to appear at each sampling date after several days of rain [27]. It seems that the concentrations of fermentable nitrogen in grape berries fluctuate during ripening and are more likely to be affected by water availability [26,28]. With the increase of grape maturity, it is interesting to note these strong positive correlations among NH_4_^+^, *α*-NH_2_ and YAN contents in grape juices, regardless of different cultivars or vintages (PM: *r =* 0.790–0.989, CH: *r =* 0.886–1.000; Table 3 and Table 4). However, this observation is not always consistent with the results of other authors [28,29].

YAN level in grape juice is vital for the development of yeasts during alcoholic fermentation and is recommended to be at least 150 mg/L in order to prevent ‘stuck’ fermentations [21]. In our study, YAN levels in CH grape juices were relatively lower than those of PM cultivar, especially in CH S1 (79 mg/L) and CH S2 (93 mg/L) grape juices at maturity level II in 2015 (Table 2). Nevertheless, no stuck fermentations occurred in our study, probably because the nitrogen content (79–333 mg/L) was sufficient for yeast growth and metabolic needs.

### 2.5. Wine Proteins

#### 2.5.1. Total Protein Concentration Determined by the Bradford Method

As shown in the Table 1 and Table 2, protein concentration varied from 11.1 to 18.8 mg/L eq. BSA in PM wines, and from 3.4 to 10.1 mg/L eq. BSA in CH wines. From grape maturity level I to III, the general trend was an increase of the total protein content in base wine, whatever the cultivar, the vintage or the pressing cycle stage. According to a previous study [30], most wine proteins come from grape berries, and some are released by yeast during alcoholic fermentation. In this study, yeast inoculation and fermentation processes were meticulously controlled under the same conditions, thus the increase in wine total protein content was largely due to the increment of grape maturity. This result was then confirmed by the observed significant correlation coefficients between grape MD and wine total protein content: *r =* 0.979–0.980 for PM cultivar and *r =* 0.859–0.973 for CH cultivar. Moreover, wine total protein content also showed significant/high correlation to other grape maturity parameters, including pH (0.909–0.993), TA (−0.904–−0.966) and MA (−0.805–−0.974).

#### 2.5.2. Wine Protein Concentration and Composition Assessed by SDS–PAGE

Electrophoretic analysis is of great interest due to its ability to determine protein concentration and assess protein fractions by their molecular weights (MWs). Concerning the MWs of the different protein bands, the accuracy was between 3–9% (generally, the higher the MW, the lower the precision), depending on the position of the protein band on the gel. For protein band quantifications, the accuracy was between 1–10% for the PM 2015, for example. In this study, SDS–PAGE was utilized to analyze the soluble proteins of PM and CH base wines.

**Figure 1:**
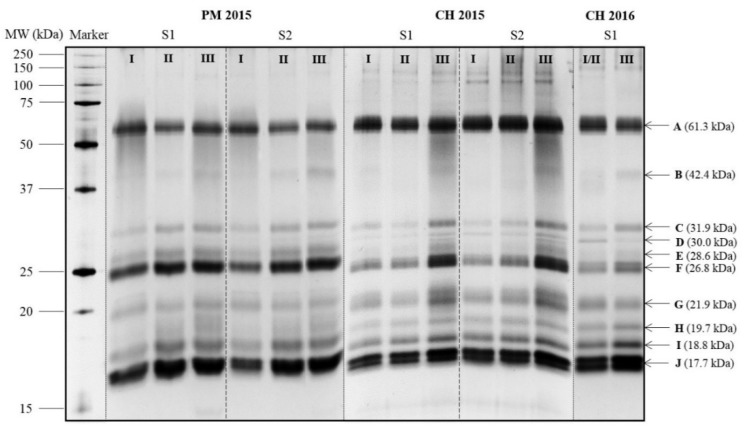
SDS–PAGE of Pinot meunier (PM) and Chardonnay (CH) Champagne base wine proteins from different grape maturity levels by silver staining.

As shown in Figure 1, the protein bands of PM and CH base wines were distributed in a wide range of MWs varying from 250 to 15 kDa, but their composition remained unchanged whatever the maturity level. Among them, 10 major bands of these proteins, named from A to J with MWs ranging from 61.3 to 17.7 kDa, were quantified. The sum of these 10 main protein bands’ contents were regarded as the total protein concentration of base wines. Total protein concentrations varied from 12.29 to 17.66 mg/L eq. 50 kDa marker in PM base wines and from 10.25 to 16.44 mg/L eq. 50 kDa marker in CH base wines (Table 1 and Table 2). The total protein contents of PM base wines were always higher than that in CH base wines, whatever the method (Table 1 and Table 2). Concerning grape maturity level from I to III, wine total protein content by SDS–PAGE increased as grape berry MD augmented (*r =* 0.799–0.967).

**Figure 2:**
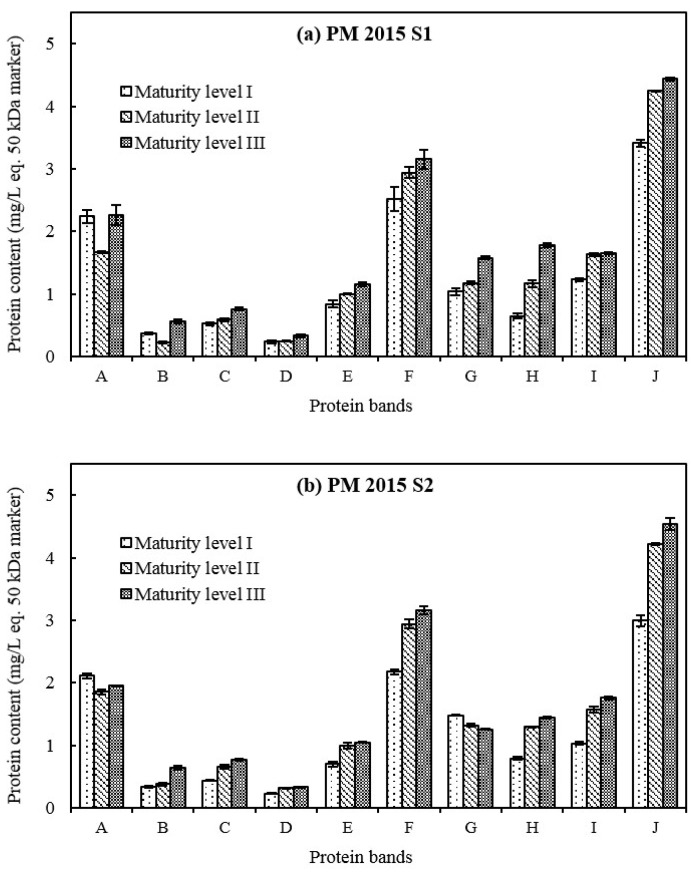
Concentrations of 10 major wine protein bands from Pinot meunier (PM) S1 (**a**) and S2 (**b**) base wines in vintage 2015. Error bars represent the standard deviation of three replicates.

**Figure 3:**
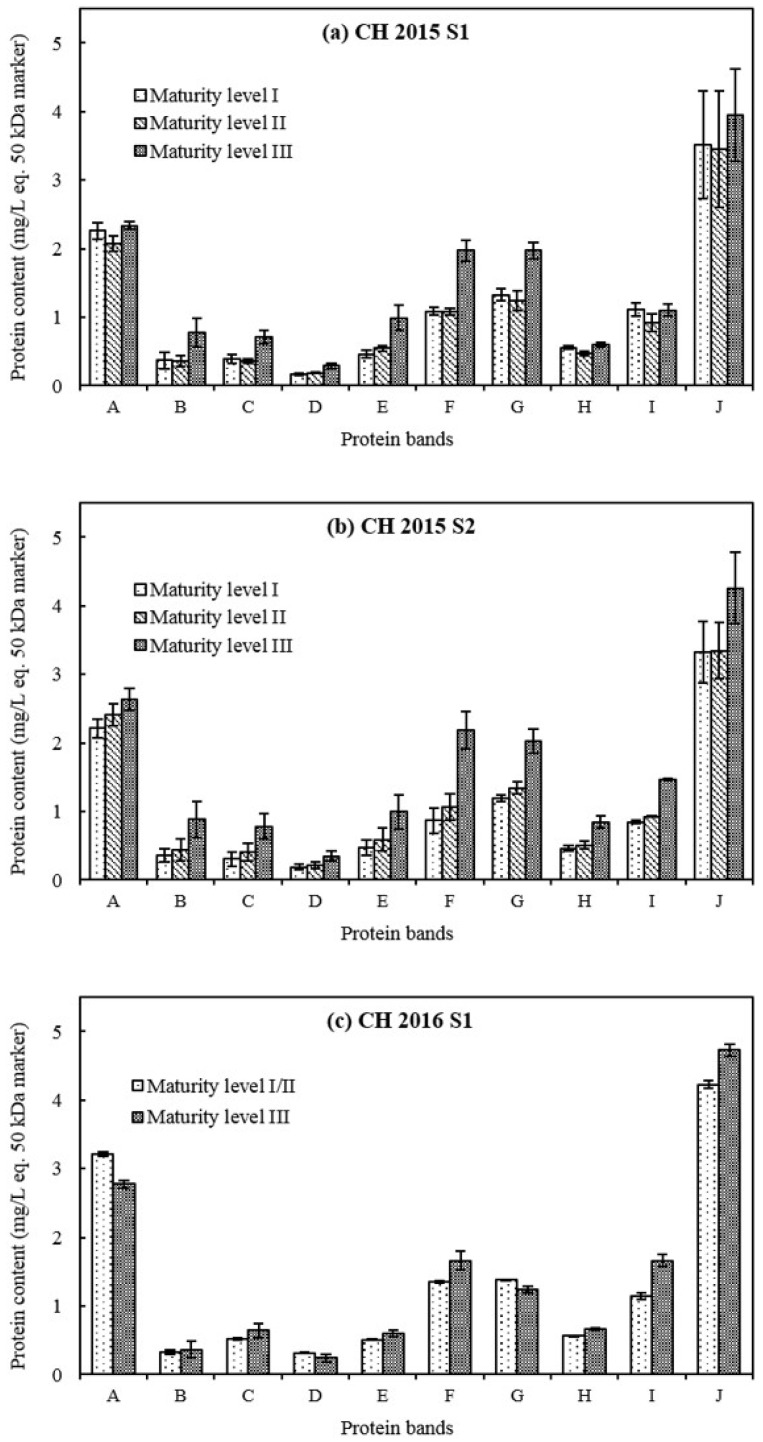
Concentrations of 10 major wine protein bands from Chardonnay (CH) S1 (**a**) and S2 (**b**) base wines in vintage 2015, and S1 (**c**) base wines in vintage 2016. Error bars represent the standard deviation of three replicates.

The three most intense protein bands (A, F and J) appeared respectively at 61.3, 26.8 and 17.7 kDa in base wines (Figure 1), representing 58–64% of wine total protein content (Figure 2 and Figure 3). The concentrations of protein bands F and J increased from grape maturity level I to III, and for most of the other protein bands, such as bands B, C, E, H and I, regardless of the cultivar, the vintage or the press fraction. Those protein fractions from band C to band J with lower MWs (approximately between 31.9 and 17.7 kDa) accounted for approximately 80% of total protein content in base wines (Figure 2 and Figure 3). Their increment in concentration mainly influenced the rise in wine total protein contents. Similar results were also reported in other varieties: Pinot noir [31] in a French cool climate region, Riesling [32] in Germany, Macabeo [11] in Spain, Nebbiolo [33] and Glera [34] in Italy, Moscatel [35] in Portugal, Syrah and Cabernet Sauvignon [36] in Brazil. These proteins might be pathogenesis-related (PR) proteins, comprising chitinases and thaumatin-like proteins (TLPs) as previously identified by a proteomic approach with a Pinot meunier wine from the 2013 vintage, in the Champagne region too [37].

There is an important protein band A (61.3 kDa), likely the grape vacuolar invertase [38,39] that was observed in previous studies with Champagne wines [7,30,37,40]. The concentration of protein band A fluctuated between 1.67–2.26 mg/L eq. 50 kDa marker for PM base wines (Figure 2) and 2.07–3.22 mg/L eq. 50 kDa marker for CH base wines (Figure 3) between grape maturity level I and III. This indicated that berry vacuolar invertase is a stable protein, which is consistent with the study of Davies and Robinson [38]. Concerning grape maturity level from I to III, the proportion of band A (grape invertase) in the wine total protein content displayed a tendency to decrease: from 17% (I) to 11% (III) for PM base wines, and from 21% (I) to 16% (III) and from 24% (I) to 19% (III) for CH base wines in 2015 and in 2016, respectively. This might be due to the increase in other lower-MW protein levels leading to an increase in total protein content in base wines.

Protein band B observed at 42.4 kDa (Figure 1) was more visible in grape maturity level III than in I and II. This protein band B originated from yeast cell wall since it was absent in the proteomic profiles of grape juices in previous studies [37,41]. Comparing grape maturity levels from I to III, the concentration of protein band B almost doubled from 0.33–0.37 to 0.56–0.88 mg/L eq. 50 kDa marker in base wines of 2015, but slightly increased from 0.33 to 0.37 mg/L eq. 50 kDa marker for CH base wines in 2016 (Figure 2 and Figure 3). It seems that the initial composition of grape juices, especially the assimilable nitrogen like *α*-NH_2_ (much lower content found in CH base wines from grape maturity level III in 2016), could influence the metabolism of yeast and then the concentration of this protein in base wines, while the yeast inoculation and fermentation processes were well controlled.

#### 2.5.3. Comparison of Total Protein Content Determined by Bradford Method and SDS–PAGE

In this study, the total protein content of base wines was firstly determined by Bradford method, then analyzed by a silver-stained SDS–PAGE, which is more sensitive and allows the study of wine proteins without any pretreatments.

The wine total protein contents determined by a modified Bradford method (3.4–18.8 mg/L eq. BSA) varied more widely than that by SDS–PAGE (10.25–17.66 mg/L eq. 50 kDa marker). This may be because only 10 major protein bands within the range from 15 to 65 kDa were calculated in total protein content for SDS–PAGE, whilst the polyaminoacids with MWs more than 3 kDa were all measured by the Bradford method [42] and regarded as total protein content. Similarly, the increment of total protein content obtained by the Bradford method (33–74%) was higher than that observed by the SDS–PAGE technique (8–60%) from grape maturity level I to III (Table 1 and Table 2). Both methods have their own characteristics and advantages: The Bradford method is faster and low cost, while SDS–PAGE could give more information concerning the protein composition. Between the two protein quantification methods, significant/high correlations were shown for both cultivars in 2015 (0.906–0.999) and good correlation for CH cultivar in 2016 (0.784). It indicates that these two quantifications could support each other.

### 2.6. Foaming Properties of Base Wines

**Figure 4:**
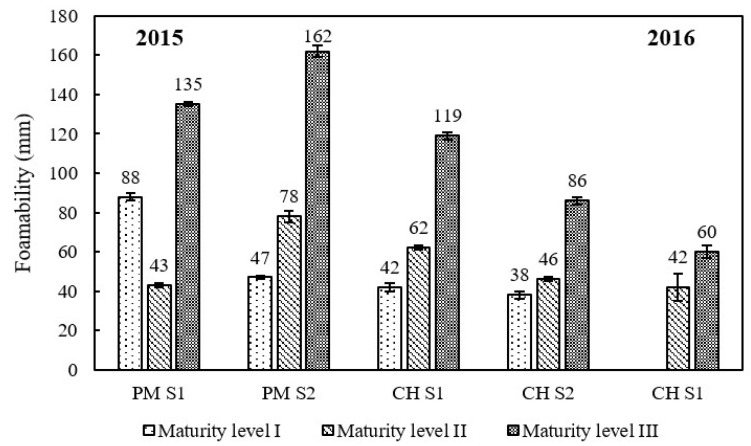
Foamability of Pinot meunier (PM) and Chardonnay (CH) base wines in vintages 2015 and 2016. Error bars represent the standard deviation of three replicates.

Base wine foamability augmented by 43–245% from grape maturity level I to III and reached the highest foam height at grape maturity level III (135 and 162 mm for PM wines, 60–119 mm for CH wines; Figure 4). High positive correlations (0.890–0.997) were found between CH wine foamability and grape berry MD. However, Esteruelas [11] reported an opposite result, that base wine foamability was higher when grapes were less ripe. Three points might explain the discrepancy between their results and ours. Firstly, in this Spanish study, the harvest was crushed before pressing and pectinolytic enzymes were used to facilitate particle flocculation and sedimentation. This procedure was quite different from that used in the present work and could have changed the composition of the grape juice, especially phenolics and antifoam compounds present on the skins. Secondly, the Spanish wines issued from the first and the second ripening stages presented small differences in alcohol contents (0.3% to 1.0% v/v) that could have slightly affected wine foamability [43]. Finally, the gluconic acid content of base wines ranged from 37 to 270 mg/L, indicating that grape health status varied between the first and second maturity level in the Spanish study. It could be that the grapes harvested first were healthy but the second ones were partially infected by *Botrytis cinerea*. In such conditions, proteins could have been partially degraded into smaller-MW fractions, which might induce a decrease in wine foamability as previous studies observed [44,45]. In the present study, the gluconic acid content of PM and CH base wines in our study ranged from 8 to 31 mg/L (Table 1 and Table 2), revealing that the health status of grape berries had been relatively well controlled. However, it is evident that berries visually considered as perfectly sound often bear traces of *Botrytis* as shown by the gluconic contents measured, especially for the last maturity level. We have never observed a grape juice without less than 6 mg/L gluconic acid in a *Cuvée* grape juice [17]. Although there was no difference in MD for PM variety between the last two grape maturity levels (II and III), PM S1 and S2 base wine foamability tripled (from 43 to 135 mm) and doubled (from 78 to 162 mm), respectively.

The increment of wine foamability might be due to the increase of protein contents (*r =* 0.611–0.851 for PM cultivar; *r =* 0.850–1.000 for CH cultivar), especially the proteins below 40 kDa, which is also reported by Esteruelas [11]. Among the 10 major protein bands, wine foamability was strongly positively correlated to the concentration of band C (*r =* 0.809), and some positive correlations (*r =* 0.613–0.696) were also found between wine foamability and seven protein bands, the bands B, D, E, F, H, I and J. Several studies indicated that these grape proteins themselves do not have foaming properties [34,39,46]. However, proteins showed a cooperative effect with glycosylated substances and contributed to the foam activity of Prosecco wines [34]. In addition, Vanrell [47] also found that a significant decrease of sparkling wine foamability was associated with the removal of grape invertase and PR proteins by bentonite treatments.

Concerning study of the foaming properties of specific macromolecular fractions/proteins [39,46], it is always extremely difficult to understand and interpret the results when using reconstituted model wines. Concerning the grape berry invertase, a strong correlation exists between this glycoprotein and the total protein content of a Chardonnay wine. There is also a high correlation between the invertase content (determined using a PTA–ELISA method) and a wine foamability [7]. Nevertheless, when the grape invertase was isolated by ion-exchange/Con A chromatography techniques, then desalted and lyophilisated, the enzyme completely lost its surface properties because of the different purification steps. For this reason, the specific contribution of a protein to the wine foamability/surface properties remains difficult to understand.

Base wine foamability also displayed strong positive (pH, sugars and PAC) and negative (TA and MA) correlations with parameters strongly changing during the maturation of the grape berries, whatever the cultivar, the year or the stage of the pressing cycle (Table 3 and Table 4). This does not mean that the MA or the compounds participating in the TA are implicated in the wine foaming properties. These correlations just indicate that the riper the grape berry, the better the base wine foamability. These results are of prime importance for base and sparkling wine producers because foam is always a parameter appreciated by sparkling wine drinkers.

### 2.7. Principle Component Analysis (PCA)

**Figure 5:**
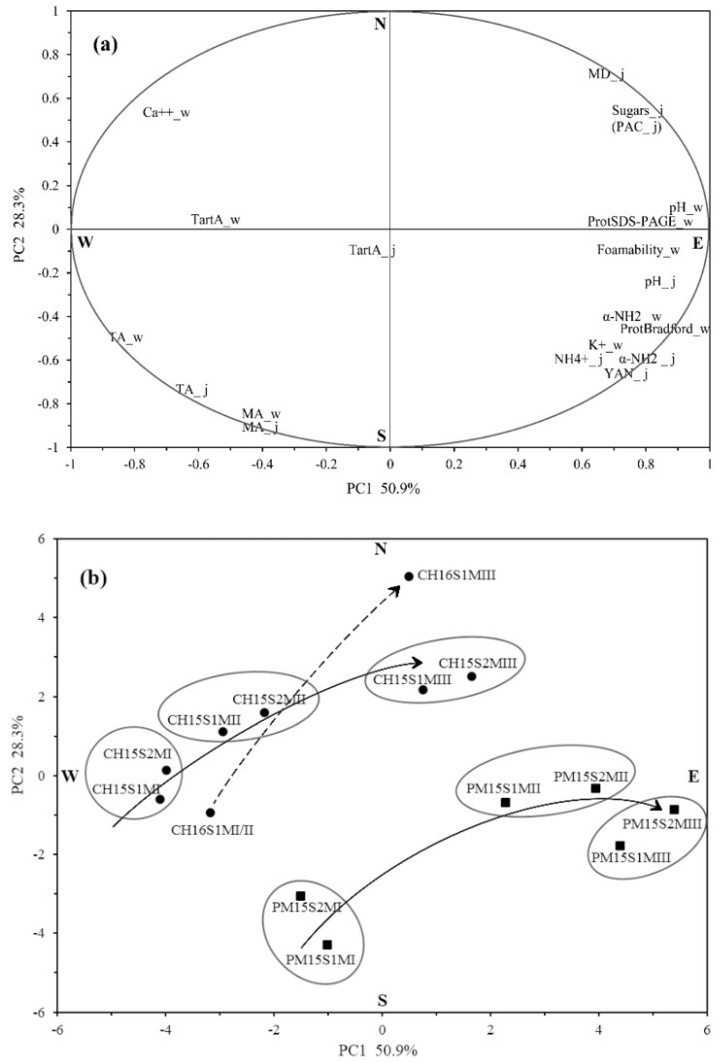
Principle component analysis applied to oenological parameters (**a**) and sample distribution (**b**) for Pinot meunier (PM) and Chardonnay (CH) grape juices and base wines.

In order to give an overall view for the results achieved above (Table 1 and Table 2 and Figure 1, Figure 2, Figure 3 and Figure 4), a PCA was applied with 20 parameters for PM and CH grape juices and base wines in vintage 2015 and 2016. The first two principal components PC1 (50.9%) and PC2 (28.3%) explained 79.2% of the total variance (Figure 5).

As can be seen in Figure 5a, grape sugars, PAC and MD were located in northeastern orientation (NE) while grape juice TA and MA were cited in the opposite direction—southwest (SW). Therefore, grape berry maturity evolved in the direction SW → NE. From Figure 5b, we could see that PM and CH samples from different maturity levels were clearly separated in this direction. In particular, the evolution of CH grape maturity level was more evident in 2016 than in 2015. The difference of maturity degree (ΔMD) for the CH S1 in 2016 was equal to 12 (ΔMD = MD_III_ − MD_I/II_ = 28 − 16 = 12). In 2015, the ΔMD was equal to 9, for the CH S1 and the PM S1. It may be related to more sunlight hours and higher temperatures during the harvest period of 2016 [48] than 2015 [27]. Base wine foamability was very closely correlated with wine protein content determined by SDS–PAGE, which confirmed their positive effects on wine foamability (Figure 5a). It is worth noting that wine foamability was found to be in the similar orientation as the grape maturity. The pH value of both grape juice and base wine showed a strong correlation with the wine foamability, indicating that pH is an interesting parameter with regards to foam. The pH is a parameter extremely easy to measure in a winery and a good indicator for someone following the maturity of a field.

The two grape varieties, CH and PM, were fairly divided in the NW → SE direction as shown in Figure 5b. In this study, it seems that PM cultivar had a better ability to foam than CH cultivar when they have similar maturity. In addition, PM was richer in grape juice NH_4_^+^, *α*-NH_2_ and YAN indexes, and base wine K^+^ and protein content, whilst CH variety was more abundant in wine Ca^++^ and had less protein.

## 3. Materials and Methods

### 3.1. Production of Juice and Wine

PM and CH grape clusters were collected from the Champagne region, France. In 2015, PM and CH grapes were hand-harvested at three different maturity levels (I, II and III) within the range of PAC from 8 to 11% v/v, which is the maturity level permitted for Champagne wine production (Table 1). In 2016, CH grapes were manually collected at two harvest times: the first grapes harvested showed sugar content and total acidity values between those of grapes at maturity level I and II in 2015, thus the first grape maturity level in 2016 was noted as I/II (Table 2); the second grape maturity level was marked as III. Unfortunately, the repetitive annual experiment for PM grapes was not undertaken in 2016 due to the high incidence of *Botrytis cinerea* at the second harvest time. The dates of harvests are given in Table 1 and Table 2. For example, II (28/08) means that the second sample was harvested the 28 August.

Healthy bunches (12 kg of vintage 2015 and 13.5 kg of vintage 2016) were hand-harvested for each stage of maturity. The bunches were pressed without destemming with a laboratory vertical wood basket-press whose capacity is 10 kg of bunches (Institut Oenologique de Champagne, Epernay, France). The ratio height/diameter of press cage is much higher than that of industrial traditional vertical presses used in wineries. Therefore, the cage was only loaded with 6 kg in 2015 (duplicate) and 4.5 kg in 2016 (triplicate).

The grape juice volume extracted from each squeeze S1 and S2 at pressing was expressed by the ratio of extracted volume/total extracted volume, at the end of the pressing cycle. These values are expressed in % (total volume obtained = 100%). The yield was the same as that applied in the Champagne region according to the local regulation: extraction of 2 liters for 3 kg of whole bunches, that is, a yield of 66% v/w. For S1, the yield was 28% and 26% for S2. No treatment with pectinolytic commercial enzymes was applied during or after pressing. The pressure applied for the first and second squeezes, S1 and S2, respectively, increased step by step to reach 1200 mbar at the end of each squeeze. The bearings of pressure were 200, 400, 600, 800, 1000 and 1200 mbar (relative pressure controlled by a waterproof pressure sensor placed under the pressing plate). At the end of S1, the bunches partially crushed were smoothly unpacked to avoid trituration and the release of particles, before beginning the second squeeze S2 at 200 mbar (same cycle as S1).

This study only presents results from S1 and S2, since they represent the high quality of Champagne called ‘*Cuvée*’. The ‘*Cuvée*’ juice, released from the grape intermediate zone as grape berries during pressing, normally contains higher concentration of sugars [49]. The free-running juice (200 mL), flowing on the surface of the grape berries as the press started, washed the cuticle waxes away, thus the negative effect of lipids on foaming properties could be eliminated [43]. In 2016, we only studied the CH grape juice and base wines from the first squeeze (S1).

After 24 h of static settling (18 °C), 100 mL of each grape juice sample was centrifuged (10 min at 17,000 *g*, 18 °C) and the supernatant was separated, filtered through a 0.45 µm HV membrane (Merck Millipore Ltd., Cork, Ireland), then directly analyzed for oenological parameters. The other part of grape juice was racked and chaptalized with sucrose to obtain an alcohol content of 11% v/v in the base wine. Alcoholic fermentation was achieved by inoculating *Saccharomyces* *cerevisiae* var. *bayanus* (IOC 18-2007 strain) at 18 °C. Once alcoholic fermentation had finished, base wines were centrifuged (15 min at 17,000 *g*, 18 °C) and the clear wines were sulfited (8 g/hL) and stabilized at 4 °C for 5 weeks. Afterwards, 100 mL of each stable wine sample was filtered through a 0.45 µm HV membrane, and then directly analyzed for oenological parameters.

### 3.2. Chemical Compositions

The analytical methods recommended by the Compendium of International Methods of Wine and Must Analysis (International Organization of Vine and Wine—O.I.V. Paris, France, 2009) were used to determine the pH, TA (g/L H_2_SO_4_), MA (g/L), TartA (g/L), sugars (g/L), K^+^ (mg/L), Ca^++^ (mg/L), PAC (% v/v), alcohol content (% v/v), YAN (mg/L), NH_4_^+^ (mg/L), *α*-NH_2_ (mg/L) and gluconic acid (mg/L) of grape juices and/or base wines. The grape berry MD was calculated as the ratio of sugars to TA.

An ATP 3000 potentiometer (ISITEC-LAB, Montauban, France) was used to determine the pH and TA. The TA was determined by NaOH titration, using bromothymol blue as an indicator. An Anton Paar DMA 35 Density Meter (Anton Paar, Courtaboeuf, France) was used to analyze sugar content, which is calculated according to the mass per volume unit. The PAC is derived from sugar content, and the result is based on the performance of yeast (16.83 g/L of sugar produces 1% volume of alcohol). The MA, TartA, gluconic acid, NH_4_^+^, *α*-NH_2_, K^+^ and Ca^++^ were determined using a UV-visible Hitachi 911 sequential analyzer (Roche Diagnostics, Meylan, France). Gluconic acid is a chemical index currently used to estimate the level of *Botrytis cinerea* infection. YAN content is the sum of NH_4_^+^ and *α*-NH_2_. The alcohol content was determined by near-infrared spectrometry with a Spectra Alyser (Zeutec GmbH, Rendsburg, Germany).

### 3.3. Wine Protein Analysis

Two different techniques, the method of Bradford and the SDS–PAGE, were used to determine the total protein concentration of base wines in this study.

1) A modified Bradford method [50] was used to avoid interferences due to ethanol and phenolic compounds. In brief, the wine protein reactivity with the Coomassie Blue Brilliant is equal to the difference between wine and ultra-filtrate reactivities with the dye reagent, respectively. Wines were ultra-filtrated with Amicon^®^ Ultra-4 (3 kDa MWCO, Merck Millipore, Tullagreen, Carrigtwohill Co. Cork, Ireland) and the ultra-filtrate was recovered. The assay was carried out as follows: 200 µL of Bradford dye reagent (Bio-Rad Laboratories GmbH, Munich, Germany) was added to 400 µL of sample (wine or ultrafiltrate) plus 400 µL of ultrapure water. Absorbance of the mixture was determined at 595 nm after 30 min of reaction. Results were expressed in mg/L equivalent to bovine serum albumin (BSA) which was used as a standard. Each value was the average of three independent measures. The standard curve coefficient of correlation was R^2^ = 0.9956 for PM wines vintage 2015, and 0.9981 and 0.9946 for CH wines vintage 2015 and 2016, respectively.

2) Wine protein compositions and quantifications were also assessed by SDS–PAGE according to the method of Laemmli [51]. The 8.3 cm × 7.3 cm dimension and 1.0-mm-thick slab gel was composed of 4% polyacrylamide (Bio-Rad Laboratories, Inc., Beijing, China) stacking gel and 13% polyacrylamide separating gel. A vertical Mini-PROTEAN^®^ III electrophoresis apparatus (Bio-Rad Laboratories S.r.l., Segrate, Italy and Bio-Rad Laboratories Singapore Pte. Ltd.) was used to run the gel at a constant voltage of 150 V until the bromophenol blue tracker dye reached the gel bottom. Wine samples were mixed with the 4x Laemmli buffer (v/v = 3:1) and 18 µL of the mixture was loaded in each well. Five-fold diluted standard proteins from 10 to 250 kDa (Precision Plus Protein ^TM^ Unstained Standards, Bio-Rad Laboratories, Inc., Hercules, US State) were used as MW markers and 1 µL was loaded. The MWs of wine proteins were calculated from the linear regression equation of log MW versus mobility. After migration, gels were silver-stained according to the protocol described by Rabilloud [52]. For each sample, the gels were carried out in triplicate. After silver nitrate coloration, the SDS–PAGE gels were scanned with a Bio-Rad Doc XR^+^ scanner and analyzed using the Image Lab software. The quantity of marker 50 kDa (74 ng/µL) was regarded as a standard for each protein band and their contents were expressed as mg/L eq. 50 kDa marker.

### 3.4. Foaming Properties

All base wines were filtered through a 0.45 µm HV membrane and stored at 18 °C overnight. Foam measurements were carried out using a DFA-100 dynamic foam analyzer (Krüss GmbH, Hamburg, Germany). The Krüss foam analyzer is a sparging procedure similar to the Mosalux equipment used in previous studies [7,8]. The main advantage is that less wine is used, only 20 mL, whereas 100 mL is required for the Mosalux. Base wine (20 mL) was poured into a glass cylinder (2 cm in diameter and 25 cm long). CO_2_ was then injected into the glass cylinder through a glass frit (pore size 40–100 µm) with a constant gas flow rate (80 mL/min) under a constant pressure (300 kPa). The foamability, corresponding to the maximum foam height reached by foam, was measured in millimeters. Each experiment was done in triplicate. For each series, all measurements were determined on the same day at 18 °C to reduce the experimental error.

### 3.5. Statistical Analysis

The Pearson’s correlation test was performed by using Microsoft Office Excel 2016 software. The correlation coefficients (*r* values, *p <* 0.05) were obtained to reveal the relationships between oenological parameters such as pH, TA, MA, TartA, sugar content, PAC, grape berry MD, K^+^, Ca^++^, YAN, NH_4_^+^ and *α*-NH_2_ for grape juices and pH, TA, MA, TartA, K^+^, Ca^++^, *α*-NH_2_, total protein content (Bradford and SDS–PAGE quantifications) and foaming characteristic of base wines. The coefficients that give a significant positive or negative correlation between two parameters were marked in green (*r >* 0.95) and in red (*r <* −0.95) in Table 3 and Table 4 (Results section). The coefficients showing a high positive or negative correlation are presented in pale green (0.95 > *r >* 0.8) and in pink (−0.95 *< r <* −0.8). Those correlation coefficients of oenological parameters (grape juice versus grape juice, grape juice versus base wine, and base wine versus base wine) were then counted.

The PCA was carried out using Scilab software (version 6.0.0; Scilab Enterprises, Rungis, France) with the Fact toolbox (INRA Montpellier, France). Except for grape juice K^+^ and Ca^++^ (lack of data), all 20 oenological parameters of grape juice and corresponding base wines were analysed by PCA, to visualize to what extent the grape maturity and chemical components of grape juice and base wine could influence the wine foaming characteristic.

## 4. Conclusions

Under global warming conditions, winemakers are concerned about how maturity affects parameters linked to grape berry maturation and maturity. For this reason, this paper studied how grape ripening in a cool climate region can affect grape juice and wine composition, specifically how it affects the foaming properties of base wines in relation to the protein content.

The main conclusion of this study is the riper the grape berry, the higher the wine protein contents and the higher the foamability of the base wines. This result confirms that proteins play a central role in sparkling wine foaming potential, and provides information for cool-climate regions producing sparkling base wines.

This research also showed that it is not possible every year to harvest disease-free bunches that provide grape juice with a PAC of between 10–10.5% v/v. Indeed, the rainy conditions at harvest time in the Champagne region often result in harvest bunches with a PAC between 8.5% and 9.5% v/v. This leads to wines with low foamability.

Further conclusions from this study are the high relationships (Pearson’s test) and positive/negative correlations (PCA test) between the base wine foam and the grape juice pH or TA. Practically, it means that it is possible to follow the optimal date to harvest, as long as the fruit remains healthy. The study of the proteins explains the changes in wine foamability. Moreover, a pH meter found in many wineries can provide more interesting information regarding foam than the sugar content (or the maturity degree).

Further investigations are underway regarding the potential relationships between the parameters studied in this work and the sensory qualities of the grape juice and finished wines.

## Figures and Tables

**Table 1:** Oenological parameters of Pinot meunier grape juices and base wines at different grape berry maturity levels.

Parameters	S1 PM 2015						S2 PM 2015					
	Grape Juice			Base Wine			Grape Juice			Base Wine		
	I (26/08)	II (04/09)	III (15/09)	I	II	III	I (26/08)	II (04/09)	III (15/09)	I	II	III
pH	2.90 ± 0.01	2.97 ± 0.01	2.96 ± 0.01	2.73 ± 0.00	2.81 ± 0.01	2.85 ± 0.00	2.92 ± 0.01	3.08 ± 0.01	3.05 ± 0.01	2.76 ± 0.00	2.88 ± 0.00	2.93 ± 0.01
TA (g/L H_2_SO_4_)	10.8 ± 0.0	7.9 ± 0.0	8.6 ± 0.0	9.8 ± 0.1	7.6 ± 0.0	7.9 ± 0.0	10.4 ± 0.0	7.4 ± 0.0	7.8 ± 0.0	9.1 ± 0.0	6.9 ± 0.1	6.8 ± 0.0
MA (g/L)	10.2 ± 0.4	7.0 ± 0.2	7.2 ± 0.3	7.0 ± 0.4	5.9 ± 0.1	5.9 ± 0.1	9.7 ± 0.4	6.7 ± 0.3	6.8 ± 0.3	6.7 ± 0.1	5.6 ± 0.0	5.3 ± 0.0
TartA (g/L)	8.2 ± 0.3	7.0 ± 0.2	9.4 ± 0.3	5.5 ± 0.2	4.4 ± 0.1	5.3 ± 0.0	8.0 ± 0.3	7.1 ± 0.2	8.9 ± 0.3	5.4 ± 0.1	3.6 ± 0.1	4.5 ± 0.0
Sugars (g/L)	133.2 ± 0.5	167.1 ± 0.5	182.9 ± 0.5	-	-	-	133.5 ± 0.5	171.9 ± 0.5	180.1 ± 0.5	-	-	-
PAC (% v/v)	7.9 ± 0.0	9.9 ± 0.0	10.9 ± 0.0	-	-	-	7.9 ± 0.0	10.2 ± 0.0	10.7 ± 0.0	-	-	-
Grape berry MD	12	21	21	-	-	-	13	23	23	-	-	-
K^+^ (mg/L)	1162 ± 84	1179 ± 85	1262 ± 91	564 ± 6	484 ± 22	603 ± 13	1381 ± 99	1517 ± 109	1464 ± 105	542 ± 45	569 ± 18	585 ± 20
Ca^++^ (mg/L)	73 ± 2	45 ± 1	61 ± 2	53 ± 1	41 ± 1	46 ± 1	86 ± 3	51 ± 2	67 ± 2	60 ± 3	45 ± 0	53 ± 4
YAN (mg/L)	303 ± 7	302 ± 7	333 ± 8	-	-	-	252 ± 6	291 ± 7	331 ± 8	-	-	-
NH₄⁺ (mg/L)	148 ± 1	142 ± 1	156 ± 2	-	-	-	119 ± 1	124 ± 1	143 ± 1	-	-	-
*α*-NH_2_ (mg/L)	155 ± 2	160 ± 2	177 ± 3	22 ± 1	21 ± 1	48 ± 5	132 ± 2	167 ± 3	188 ± 3	18 ± 1	23 ± 1	42 ± 5
Prot content by Bradford (mg/L eq. BSA)	-	-	-	12.7 ± 1.6	17.4 ± 0.1	18.7 ± 0.2	-	-	-	11.1 ± 1.4	17.2 ± 0.4	18.8 ± 0.1
Prot content by SDS–PAGE (mg/L eq. 50 kDa marker)	-	-	-	13.07 ± 0.40	14.88 ± 0.10	17.66 ± 0.27	-	-	-	12.29 ± 0.06	15.55 ± 0.11	16.91 ± 0.02
Gluconic acid (mg/L)	-	-	-	15 ± 0	31 ± 2	18 ± 0	-	-	-	16 ± 1	20 ± 2	15 ± 1
Alcohol (% v/v)	-	-	-	11.0 ± 0.1	11.2 ± 0.0	10.9 ± 0.1	-	-	-	11.0 ± 0.0	11.2 ± 0.0	11.1 ± 0.0

TA: total acidity. MD: maturity degree, as the ratio of sugars to TA. PAC: the potential alcohol content of grape berries. MA: malic acid. TartA: tartaric acid. K^+^: potassium. Ca^++^: calcium. YAN: assimilable nitrogen content. NH_4_⁺: ammonium nitrogen. *α*-NH_2_: *α*-amino nitrogen. Prot: protein. SDS–PAGE: sodium dodecyl sulfate–polyacrylamide gel electrophoresis. S1 and S2: squeeze 1 and squeeze 2. PM: Pinot meunier. I, II, III: maturity levels. All data are given by the mean ± standard deviation of duplicate.

**Table 2:** Oenological parameters of Chardonnay grape juices and base wines at different grape berry maturity levels.

Parameters	S1 CH 2015						S2 CH 2015						S1 CH 2016			
	Grape Juice			Base Wine			Grape Juice			Base Wine			Grape Juice		Base Wine	
	I (29/08)	II (07/09)	III (17/09)	I	II	III	I (29/08)	II (07/09)	III (17/09)	I	II	III	I/II (13/09)	III (26/09)	I/II	III
pH	2.78 ± 0.1	2.78 ± 0.1	2.81 ± 0.1	2.64 ± 0.01	2.64 ± 0.00	2.80 ± 0.00	2.80 ± 0.1	2.80 ± 0.1	2.88 ± 0.1	2.64 ± 0.00	2.71 ± 0.03	2.84 ± 0.00	2.86 ± 0.03	2.93 ± 0.01	2.70 ± 0.06	2.81 ± 0.01
TA (g/L H_2_SO_4_)	9.9 ± 0.0	9.3 ± 0.0	7.4 ± 0.0	9.5 ± 0.0	8.9 ± 0.0	7.5 ± 0.1	9.3 ± 0.0	8.4 ± 0.0	6.9 ± 0.0	9.0 ± 0.0	8.4 ± 0.0	7.2 ± 0.1	9.9 ± 0.6	6.8 ± 0.2	9.8 ± 0.5	7.2 ± 0.1
MA (g/L)	8.3 ± 0.3	7.0 ± 0.3	5.2 ± 0.2	6.5 ± 0.2	5.8 ± 0.0	4.3 ± 0.0	7.7 ± 0.3	6.7 ± 0.3	5.0 ± 0.2	6.1 ± 0.0	5.5 ± 0.0	4.1 ± 0.0	9.2 ± 0.7	3.9 ± 0.3	7.0 ± 0.7	3.4 ± 0.1
TartA (g/L)	8.2 ± 0.3	8.6 ± 0.3	8.6 ± 0.3	5.8 ± 0.2	4.9 ± 0.1	6.1 ± 0.5	8.1 ± 0.3	8.1 ± 0.3	8.4 ± 0.3	5.4 ± 0.4	5.0 ± 0.0	5.6 ± 0.0	9.4 ± 0.1	7.9 ± 0.1	6.8 ± 0.1	5.4 ± 0.2
Sugars (g/L)	134.5 ± 0.5	157.8 ± 0.5	167.8 ± 0.5	-	-	-	135.0 ± 0.5	157.3 ± 0.5	170.6 ± 0.5	-	-	-	154.8 ± 4.5	186.8 ± 1.6	-	-
PAC (% v/v)	8.0 ± 0.0	9.4 ± 0.0	10.0 ± 0.0	-	-	-	8.0 ± 0.0	9.4 ± 0.0	10.1 ± 0.0	-	-	-	9.1 ± 0.2	11.1 ± 0.1	-	-
Grape berry MD	14	17	23	-	-	-	15	19	25	-	-	-	16	28	-	-
K^+^ (mg/L)	868 ± 62	838 ± 60	891 ± 64	427 ± 8	458 ± 7	524 ± 30	854 ± 61	879 ± 63	932 ± 67	402 ± 3	482 ± 13	540 ± 18	1160 ± 15	-	441 ± 51	354 ± 59
Ca^++^ (mg/L)	126 ± 4	108 ± 3	89 ± 3	99 ± 0	81 ± 1	82 ± 1	124 ± 4	94 ± 3	86 ± 3	99 ± 2	80 ± 3	77 ± 3	112 ± 6	-	110 ± 6	108 ± 7
YAN (mg/L)	139 ± 3	79 ± 2	177 ± 4	-	-	-	134 ± 3	93 ± 2	185 ± 4	-	-	-	218 ± 16	131 ± 21	-	-
NH₄⁺ (mg/L)	53 ± 1	24 ± 0	71 ± 1	-	-	-	53 ± 1	29 ± 0	69 ± 1	-	-	-	128 ± 12	82 ± 10	-	-
*α*-NH_2_ (mg/L)	86 ± 1	55 ± 1	106 ± 2	12 ± 0	12 ± 0	16 ± 1	81 ± 1	65 ± 1	116 ± 2	11 ± 0	12 ± 0	16 ± 1	71 ± 2	49 ± 12	11 ± 1	9 ± 1
Prot content by Bradford (mg/L eq. BSA)	-	-	-	7.6 ± 0.8	6.9 ± 0.1	10.1 ± 0.8	-	-	-	5.3 ± 0.4	5.8 ± 0.5	9.2 ± 0.4	-	-	3.4 ± 0.1	5.3 ± 0.2
Prot content by SDS–PAGE (mg/L eq. 50 kDa marker)	-	-	-	11.25 ± 0.86	10.69 ± 1.14	14.70 ± 0.56	-	-	-	10.25 ± 0.50	11.26 ± 0.58	16.44 ± 0.92	-	-	13.56 ± 0.07	14.59 ± 0.57
Gluconic acid (mg/L)	-	-	-	13 ± 2	9 ± 1	8 ± 1	-	-	-	12 ± 1	9 ± 1	9 ± 1	-	-	17 ± 4	12 ± 1
Alcohol (% v/v)	-	-	-	10.5 ± 0.0	11.1 ± 0.0	11.0 ± 0.1	-	-	-	10.6 ± 0.0	11.1 ± 0.0	11.1 ± 0.1	-	-	11.0 ± 0.1	11.1 ± 0.1

TA: total acidity. MD: maturity degree, as the ratio of sugars to TA. PAC: the potential alcohol content of grape berries. MA: malic acid. TartA: tartaric acid. K^+^: potassium. Ca^++^: calcium. YAN: assimilable nitrogen content. NH_4_⁺: ammonium nitrogen. *α*-NH_2_: *α*-amino nitrogen. Prot: protein. SDS–PAGE: sodium dodecyl sulfate–polyacrylamide gel electrophoresis. S1 and S2: squeeze 1 and squeeze 2. CH: Chardonnay. I, II, III: maturity levels. All data are given by the mean ± standard deviation of duplicate (2015) or triplicate (2016).

**Table 3:** Pearson test correlation coefficients (*r*) among the oenological parameters of Pinot meunier grape juices and base wines (*p <* 0.05).

Parameters		Grape Juices												Base wines									
		pH	TA (g/L H₂SO₄)	MA (g/L)	TartA (g/L)	Sugars (g/L)	PAC (% v/v)	Grape berry MD	K^+^ (mg/L)	Ca^++^ (mg/L)	YAN (mg/L)	NH_4_^+^ (mg/L)	α-NH_2_ (mg/L)	pH	TA (g/L H₂SO₄)	MA (g/L)	TartA (g/L)	K^+^ (mg/L)	Ca^++^ (mg/L)	α-NH_2_ (mg/L)	Prot Content (mg/L eq. BSA)	Prot Content (mg/L eq. 50 kDa marker)	Foamability (mm)
Grape juices	pH	1																					
	TA (g/L H₂SO₄)	−0.995	1															r > 0.95 : significant positive correlation			r < −0.95 : significant negative correlation		
		−0.999																					
	MA (g/L)	−0.997	0.984	1														0.95 > r > 0.80 : high positive correlation			‒0.95 < r < −0.80 : high negative correlation		
		−0.989	0.996																				
	TartA (g/L)	−0.132	0.231	0.056	1																		
		−0.176	0.123	0.029																			
	Sugars (g/L)	0.901	−0.853	−0.932	0.311	1																	
		0.942	−0.959	−0.981	0.165																		
	PAC (% v/v)	0.893	−0.844	−0.925	0.327	1.000	1																
		0.941	−0.958	−0.981	0.167	1.000																	
	Grapeberry MD	0.991	−0.973	−0.998	0.000	0.950	0.945	1															
		0.984	−0.992	−1.000	0.000	0.986	0.986																
	K^+^ (mg/L)	0.523	−0.435	−0.587	0.776	0.841	0.850	0.631	1														
		0.976	−0.963	−0.933	−0.387	0.846	0.845	0.922															
	Ca^++^ (mg/L)	−0.890	0.931	0.852	0.569	−0.604	−0.590	−0.822	−0.077	1													
		−0.956	0.939	0.903	0.457	−0.802	−0.801	−0.890	−0.997														
	YAN (mg/L)	0.355	−0.259	−0.425	0.880	0.725	0.737	0.475	0.982	0.110	1												
		0.760	−0.794	−0.847	0.506	0.934	0.935	0.862	0.600	−0.536													
	NH_4_^+^ (mg/L)	−0.050	0.151	−0.026	0.997	0.388	0.404	0.082	0.825	0.500	0.916	1											
		0.518	−0.564	−0.639	0.750	0.776	0.777	0.661	0.320	−0.246	0.950												
	α−NH_2_ (mg/L)	0.573	−0.487	−0.634	0.737	0.872	0.880	0.676	0.998	−0.136	0.970	0.790	1										
		0.849	−0.876	−0.917	0.371	0.977	0.978	0.929	0.713	−0.657	0.989	0.892											
Base wines	pH	0.893	−0.844	−0.925	0.327	1.000	1.000	0.945	0.850	−0.590	0.737	0.404	0.880	1									
		0.893	−0.916	−0.949	0.286	0.992	0.993	0.958	0.773	−0.722	0.971	0.848	0.996										
	TA (g/LH₂SO₄)	−1.000	0.994	0.998	0.126	−0.904	−0.896	−0.992	−0.529	0.887	−0.361	0.044	−0.578	−0.896	1								
		−0.977	0.987	0.998	−0.038	−0.992	−0.992	−0.999	−0.907	0.871	−0.881	−0.690	−0.942	−0.968									
	MA (g/L)	−0.991	0.973	0.998	0.000	−0.950	−0.945	−1.000	−0.631	0.822	−0.475	−0.082	−0.676	−0.945	0.992	1							
		−0.928	0.947	0.973	−0.203	−0.999	−0.999	−0.979	−0.824	0.778	−0.947	−0.800	−0.985	−0.996	0.986								
	TartA (g/L)	−0.736	0.801	0.682	0.768	−0.370	−0.354	−0.640	0.191	0.964	0.371	0.713	0.133	−0.354	0.732	0.640	1						
		−0.941	0.921	0.880	0.500	−0.772	−0.770	−0.866	−0.992	0.999	−0.494	−0.197	−0.619	−0.687	0.846	0.746							
	K^+^ (mg/L)	−0.323	0.417	0.250	0.981	0.120	0.137	−0.195	0.638	0.719	0.770	0.961	0.591	0.137	0.317	0.195	0.878	1					
		0.850	−0.878	−0.919	0.368	0.978	0.978	0.930	0.715	−0.659	0.988	0.891	1.000	0.996	−0.943	−0.985	−0.621						
	Ca^++^ (mg/L)	−0.957	0.981	0.932	0.415	−0.736	−0.724	−0.910	−0.253	0.984	−0.067	0.339	−0.309	−0.724	0.955	0.910	0.901	0.584	1				
		−0.927	0.905	0.861	0.533	−0.747	−0.745	−0.846	−0.986	0.996	−0.460	−0.160	−0.588	−0.658	0.825	0.720	0.999	−0.591					
	α−NH_2_ (mg/L)	0.351	−0.255	−0.422	0.882	0.722	0.734	0.471	0.982	0.115	1.000	0.918	0.969	0.734	−0.357	−0.471	0.375	0.773	−0.063	1			
		0.518	−0.564	−0.639	0.750	0.776	0.777	0.661	0.320	−0.246	0.950	1.000	0.892	0.848	−0.690	−0.800	−0.197	0.891	−0.160				
	Prot content (mg/Leq.BSA)	0.943	−0.904	−0.966	0.206	0.994	0.992	0.979	0.777	−0.687	0.646	0.286	0.813	0.992	−0.945	−0.979	−0.469	0.011	−0.805	0.643	1		
		0.930	−0.949	−0.974	0.197	0.999	1.000	0.980	0.828	−0.782	0.945	0.796	0.983	0.996	−0.987	−1.000	−0.751	0.984	−0.725	0.796			
	Prot content (mg/Leq.50kDamarker)	0.713	−0.638	−0.764	0.601	0.946	0.952	0.799	0.971	−0.314	0.909	0.665	0.983	0.952	−0.717	−0.799	−0.050	0.434	−0.478	0.907	0.906	1	
		0.893	−0.916	−0.949	0.286	0.992	0.993	0.958	0.773	−0.722	0.971	0.848	0.996	1.000	−0.968	−0.996	−0.687	0.996	−0.658	0.848	0.996		
	Foamability (mm)	−0.120	0.219	0.043	1.000	0.323	0.339	0.013	0.783	0.559	0.886	0.998	0.745	0.339	0.113	−0.013	0.760	0.978	0.403	0.888	0.218	0.611	1
		0.523	−0.568	−0.643	0.747	0.779	0.780	0.665	0.324	−0.250	0.951	1.000	0.895	0.851	−0.693	−0.803	−0.202	0.893	−0.164	1.000	0.799	0.851	

The correlation coefficients represent PM S1 (above) and PM S2 (below) in each grid. PM: Pinot meunier. S1 and S2: squeeze 1 and squeeze 2.

**Table 4:** Pearson test correlation coefficients (*r*) among the oenological parameters of Chardonnay grape juices and base wines (*p <* 0.05).

Parameters		Grape Juices												Base Wines									
		pH	TA (g/L H₂SO₄)	MA (g/L)	TartA (g/L)	Sugars (g/L)	PAC(% v/v)	Grape berry MD	K^+^ (mg/L)	Ca^++^ (mg/L)	YAN (mg/L)	NH_4_^+^ (mg/L)	α-NH_2_ (mg/L)	pH	TA (g/L H₂SO₄)	MA (g/L)	TartA (g/L)	K^+^ (mg/L)	Ca^++^ (mg/L)	α-NH_2_ (mg/L)	Prot content (mg/L eq. BSA)	Prot content (mg/L eq. 50 kDa marker)	Foamability (mm)
Grape juices	pH	1																					
																				
																				
	TA (g/L H₂SO₄)	−0.973	1														r > 0.95 : significant positive correlation				r < −0.95 : significant negative correlation		
		−0.929																					
		−0.947																					
	MA (g/L)	−0.909	0.980	1													0.95 > r > 0.80 : high positive correlation				‒0.95 < r < −0.80 : high negative correlation		
		−0.931	1.000																				
		−0.903	0.990																				
	TartA (g/L)	0.500	−0.686	−0.816	1																		
		1.000	−0.929	−0.931																			
		−0.905	0.955	0.968																			
	Sugars (g/L)	0.732	−0.869	−0.949	0.956	1																	
		0.785	−0.959	−0.957	0.785																		
		0.905	−0.985	−0.995	−0.959																		
	PAC (% v/v)	0.730	−0.867	−0.949	0.957	1.000	1																
		0.756	−0.945	−0.943	0.756	0.999																	
		0.910	−0.984	−0.995	−0.968	0.999																	
	Grape berry MD	0.945	−0.995	−0.995	0.756	0.914	0.913	1															
		0.918	−1.000	−0.999	0.918	0.966	0.954																
		0.932	−0.995	−0.997	−0.974	0.994	0.995																
	K^+^ (mg/L)	0.826	−0.674	−0.514	−0.076	0.219	0.217	0.595	1														
		0.949	−0.998	−0.998	0.949	0.940	0.923	0.996															
		-	-	-	-	-	-	-															
	Ca^++^ (mg/L)	−0.874	0.962	0.997	−0.858	−0.971	−0.970	−0.985	−0.447	1													
		−0.663	0.893	0.891	−0.663	−0.984	−0.991	−0.906	−0.864														
		-	-	-	-	-	-	-	-														
	YAN (mg/L)	0.795	−0.634	−0.468	−0.129	0.167	0.165	0.552	0.999	−0.399	1												
		0.896	−0.667	−0.670	0.896	0.427	0.386	0.645	0.711	−0.261													
		−0.897	0.947	0.947	0.897	−0.972	−0.970	−0.951	-	-													
	NH_4_^+^ (mg/L)	0.791	−0.630	−0.464	−0.134	0.162	0.159	0.548	0.998	−0.394	1.000	1											
		0.803	−0.524	−0.529	0.803	0.261	0.217	0.500	0.575	−0.086	0.984												
		−0.955	0.952	0.925	0.897	−0.945	−0.946	−0.942	-	-	0.976												
	α-NH_2_ (mg/L)	0.798	−0.638	−0.473	−0.124	0.172	0.170	0.556	0.999	−0.403	1.000	1.000	1										
		0.952	−0.770	−0.773	0.952	0.557	0.519	0.752	0.807	−0.401	0.989	0.947											
		−0.780	0.807	0.826	0.794	−0.875	−0.876	−0.831	-	-	0.942	0.886											
Base wines	pH	1.000	−0.979	−0.920	0.524	0.750	0.748	0.954	0.810	−0.887	0.778	0.774	0.781	1									
		0.935	−1.000	−1.000	0.935	0.953	0.939	0.999	0.999	−0.885	0.680	0.540	0.781										
		0.990	−0.910	−0.848	−0.846	0.845	0.850	0.882	-	-	−0.839	−0.921	−0.699										
	TA (g/L H₂SO₄)	−0.956	0.998	0.991	−0.731	−0.899	−0.898	−0.999	−0.624	0.978	−0.582	−0.578	−0.586	−0.964	1								
		−0.948	0.998	0.999	−0.948	−0.941	−0.925	−0.996	−1.000	0.866	−0.708	−0.572	−0.805	−0.999									
		−0.949	0.999	0.989	0.960	−0.987	−0.987	−0.995	-	-	0.954	0.961	0.819	−0.910									
	MA (g/L)	−0.948	0.996	0.994	−0.750	−0.911	−0.910	−1.000	−0.602	0.983	−0.559	−0.555	−0.563	−0.956	1.000	1							
		−0.948	0.998	0.999	−0.948	−0.941	−0.925	−0.996	−1.000	0.867	−0.707	−0.571	−0.805	−0.999	1.000								
		−0.932	0.999	0.994	0.955	−0.991	−0.990	−0.996	-	-	0.956	0.951	0.821	−0.888	0.998								
	TartA (g/L)	0.693	−0.509	−0.329	−0.277	0.016	0.013	0.419	0.979	−0.255	0.988	0.989	0.988	0.673	−0.452	−0.427	1						
		0.693	−0.376	−0.381	0.693	0.097	0.052	0.350	0.432	0.080	0.942	0.986	0.881	0.393	−0.428	−0.428							
		−0.904	0.966	0.974	0.957	−0.987	−0.989	−0.977	-	-	0.982	0.967	0.900	−0.842	0.975	0.974							
	K^+^ (mg/L)	0.951	−0.997	−0.993	0.743	0.906	0.905	1.000	0.611	−0.981	0.568	0.563	0.572	0.959	−1.000	−1.000	0.437	1					
		0.818	−0.973	−0.972	0.818	0.998	0.995	0.979	0.957	−0.973	0.476	0.313	0.602	0.969	−0.958	−0.958	0.152						
		−0.640	0.736	0.727	0.619	−0.769	−0.755	−0.719	-	-	0.859	0.831	0.784	−0.594	0.750	0.757	0.811						
	Ca^++^ (mg/L)	−0.457	0.649	0.786	−0.999	−0.941	−0.941	−0.723	0.125	0.832	0.177	0.183	0.172	−0.481	0.697	0.717	0.324	−0.709	1				
		−0.605	0.857	0.855	−0.605	−0.968	−0.979	−0.872	−0.824	0.997	−0.188	−0.011	−0.332	−0.848	0.827	0.827	0.154	−0.953					
		0.120	0.125	0.215	0.254	−0.153	−0.155	−0.165	-	-	−0.032	−0.103	−0.170	0.180	0.121	0.147	0.117	−0.056					
	α-NH_2_ (mg/L)	1.000	−0.973	−0.909	0.500	0.732	0.730	0.945	0.826	−0.874	0.795	0.791	0.798	1.000	−0.956	−0.948	0.693	0.951	−0.457	1			
		0.977	−0.986	−0.987	0.977	0.898	0.877	0.981	0.994	−0.806	0.781	0.659	0.865	0.989	−0.994	−0.994	0.525	0.921	−0.760				
		−0.584	0.698	0.728	0.597	−0.779	−0.765	−0.711	-	-	0.860	0.751	0.901	−0.505	0.701	0.723	0.773	0.846	−0.137				
	Prot content (mg/L eq. BSA)	0.979	−0.907	−0.805	0.314	0.578	0.576	0.859	0.923	−0.757	0.901	0.899	0.903	0.973	−0.877	−0.864	0.825	0.869	−0.267	0.979	1		
		0.993	−0.966	−0.967	0.993	0.852	0.827	0.958	0.980	−0.746	0.837	0.728	0.909	0.970	−0.979	−0.979	0.604	0.879	−0.694	0.995			
		0.909	−0.957	−0.967	−0.993	0.966	0.974	0.973	-	-	−0.925	−0.929	−0.827	0.849	−0.967	−0.960	−0.979	−0.695	−0.216	−0.637			
	Prot content (mg/L eq. 50 kDa marker)	0.992	−0.935	−0.847	0.384	0.638	0.636	0.895	0.891	−0.804	0.866	0.864	0.869	0.988	−0.911	−0.899	0.780	0.903	−0.338	0.992	0.997	1	
		0.988	−0.974	−0.975	0.988	0.870	0.847	0.967	0.986	−0.769	0.818	0.703	0.894	0.978	−0.985	−0.985	0.576	0.896	−0.719	0.998	0.999		
		0.823	−0.855	−0.863	−0.834	0.844	0.847	0.867	-	-	−0.759	−0.729	−0.681	0.786	−0.839	−0.846	−0.766	−0.363	−0.088	−0.573	0.784		
	Foamability (mm)	0.968	−1.000	−0.984	0.701	0.879	0.878	0.997	0.658	−0.968	0.617	0.613	0.621	0.975	−0.999	−0.997	0.491	0.998	−0.665	0.968	0.898	0.928	1
		0.988	−0.975	−0.976	0.988	0.872	0.849	0.968	0.987	−0.771	0.816	0.700	0.893	0.979	−0.986	−0.986	0.573	0.897	−0.721	0.998	0.999	1.000	
		0.703	−0.845	−0.910	−0.912	0.898	0.903	0.890	-	-	−0.805	−0.722	−0.757	0.608	−0.844	−0.860	−0.861	−0.511	−0.434	−0.656	0.885	0.850	

The correlation coefficients represent CH S1 2015 (above), CH S2 2015 (middle) and CH S1 2016 (below) in each grid. CH: Chardonnay. S1 and S2: squeeze 1 and squeeze 2.
